# Dietary Intake Influences Adult Fertility and Offspring Fitness in Zebrafish

**DOI:** 10.1371/journal.pone.0166394

**Published:** 2016-11-21

**Authors:** Trent Newman, Noel Jhinku, Michael Meier, Julia Horsfield

**Affiliations:** 1 Department of Pathology, Dunedin School of Medicine, PO Box 913, University of Otago, Dunedin, 9054, New Zealand; 2 Gravida: National Centre for Growth and Development, University of Auckland, Auckland, New Zealand; 3 Maurice Wilkins Centre for Molecular Biodiscovery, University of Auckland, Auckland, New Zealand; University of Vienna, AUSTRIA

## Abstract

The burden of malnutrition, including both over- and undernutrition, is a major public health concern. Here we used a zebrafish model of diet-induced obesity to analyze the impact of dietary intake on fertility and the phenotype of the next generation. Over an eight-week period, one group received 60 mg of food each day (60 mg arm), while another received 5 mg (5 mg arm). At the end of the diet, the body mass index of the 60 mg arm was 1.5 fold greater than the 5 mg arm. The intervention also had a marked impact on fertility; breeding success and egg production in the 60 mg arm were increased 2.1- and 6.2-fold compared to the 5 mg arm, respectively. Transcriptome analysis of eggs revealed that transcripts involved in metabolic biological processes differed according to dietary intake. The progeny from the differentially fed fish were more likely to survive when the parents had access to more food. An intergenerational crossover study revealed that while parental diet did not influence weight gain in the offspring, the progeny of well-fed parents had increased levels of physical activity when exposed again to high nutrient availability. We conclude that dietary intake has an important influence on fertility and the subsequent fitness of offspring, even prior to breeding.

## Introduction

Development and survival of animals (including humans) depends on environmental conditions. But, in addition to the environment present during development, we should also consider the environment to which the parents are exposed. One of the most important factors in the environment is the availability of food. In 2008, it was estimated that there were 915 million undernourished people in the world [1]. However, in that same year there was also estimated to be 1046 million adults that were overweight [2]. For parents, the availability of nutrients can have a strong influence on the health and development of their offspring, even before pregnancy.

Parental under- and overnutrition can reprogram the development of the next generation and alter their risk of disease [3]. One study concluded that parental obesity more than doubled the risk of adult obesity in the children [4]. The biological children of obese parents that were adopted into new families still became obese as adults, suggesting that the family environment cannot overcome parental effects [5]. As a result, much attention has been given to the role that an abnormal in utero environment could play in programming fetal development [6].

Conceptual and empirical advances now support the possibility that traits acquired by parents might be inherited by the children, an idea that was rejected by classical genetics [7]. There are now several epidemiological studies showing that environmental exposures can have transgenerational consequences. A notable study looked at historical longitudinal cohort data and observed that the paternal grandfather’s food supply was associated with mortality in the grandsons; also, the paternal grandmother’s food supply was associated with mortality among the granddaughters [8]. There has been intense interest in epigenetic modifiers (such as DNA methylation, chromatin structure and small RNAs), that can provide long-term changes in gene expression [9]. It is thought that the inheritance of environmentally-induced epigenetic changes via the gametes could provide a mechanism for how transgenerational phenotypes arise [10].

Animal models allow prospective studies on the developmental consequences of environmental exposures in a way not possible in humans. Many studies on transgenerational epigenetic inheritance have been performed in rodents; indeed, some of the classic studies on obesity, epigenetics, and inheritance were conducted using the agouti viable yellow (A^vy^) mice [11]. However, the evidence now suggests that diet [12], toxic exposures [13], traumatic experiences [14], and transient exposure to an altered genetic background [15] can all have a transgenerational impact. Recently, the use of surrogacy has been effective in providing insight into disease inheritance via the gametes [16]; however, studies on transgenerational inheritance would benefit from an animal model with external fertilization. Zebrafish are well suited for studying development due to the ease with which they can be bred, the external fertilization of their eggs, and the number of eggs they produce. Recently, researchers have recognized the possibility of using zebrafish to model a range of human metabolic diseases, including obesity [17].

Like humans, fish carefully balance their energy intake, utilization and storage [17]. The major deposits of white adipose tissue in zebrafish adults are pancreatic, esophageal, visceral, subcutaneous, and cranial [17,18]. Models of zebrafish obesity have been described that derive from constitutive *akt* expression [19], *agrp* overexpression [20], or a high-calorie diet [21]. Zebrafish subjected to diet-induced obesity developed by Oka et al. 2010 were found to have a higher body mass index, hypertriglyceridemia and hepatosteatosis, compared with calorie-restricted control zebrafish [21]. Oka et al. also showed that transcriptional changes in the visceral adipose tissue of their obese zebrafish were similar to gene expression changes found in mammalian obesity [21]. The group subsequently went on to show that interventions, including Campari tomato [22], green tea extract [23], and Yuzu peel [24], could rescue the pathology observed in their model.

In this study, we adapted the model established by Oka et al. to look at the effect of parental nutrition on the health of the offspring. However, early on we found that the fish on the control diet showed signs of undernutrition, rather than maintaining their weight on this diet. We therefore refer to the dietary treatments in our experiment by the amount of food given to the fish, rather than designating treatment arms as control and overfed. Nonetheless, we found that the different dietary regimes had a notable impact on fertility, egg quality, and the phenotype of the offspring.

## Methods

### Animals

Zebrafish research was approved by the University of Otago Animal Ethics Committee. Mature zebrafish were maintained in 3.5 L tanks on a Palletized Centralized Life Support System (Tecniplast). The water in this recirculating system was pumped through mechanical filtration, charcoal filtration, and UV-treatment; and 10% of the water was replaced every hour. The water was kept at 26–30°C, with pH 7.6–8.0 and a conductivity of 300–600 μS. The facility environment maintained a 14-hour light and 10-hour dark circadian cycle. Water quality parameters were automatically measured and adjusted, and remained within acceptable limits for the duration of the study.

Three groups of the ABPS in-house wild type line (derived from an AB line originally obtained from ZIRC) were used. Each group was born on a different day; at the time of the dietary intervention, the groups were aged eight (group 1), seven (group 2), and six (group 3) months post fertilization (mpf). Nutrition prior to the diet consisted of a dry feed twice a day of ZM zebrafish food (Zebrafish Management Ltd.) with the grade dependent on zebrafish age. ZM000 was given up until 14 days post fertilization (dpf), ZM100 was given between 15 and 35 dpf, ZM200 was given between 36 and 90 dpf, and ZM300/400 was given after 90 dpf. A live feed was given once a day; fry were fed rotifer until 50 dpf and then given *Artemia* throughout adulthood.

### Dietary intervention

The fish from each group were randomly separated into two treatment arms: a 5 mg food per fish arm (one meal) and a 60 mg food per fish arm (three 20 mg meals), based on the Oka model [21]. There were three tanks within each treatment arm (Table 1) containing 20 fish in groups 2 and 3, and 12 fish in group 1 (due to fertility screening, below).

**Table 1:** F_0_ groups. The number of male and female fish used in each F_0_ treatment arm. All the fish were of the same strain, with the groups born a month apart from one another. We aimed to have 20 fertile fish in three tanks within each treatment arm in order to be able to detect a breeding odds ratio of 0.4 with 75% power [25]. On average there were 20 fish (5.7 fish/L) in each tank for group 2 and 3. In group 1, fertility screening only allowed 12 fish (3.4 fish/L) to be allocated into each tank. Tank 3 in group 1 has 10 fish due to mortality prior to the intervention.

Arm	Sex	Group 1			Group 2			Group 3		
		Tank 1	Tank 2	Tank 3	Tank 1	Tank 2	Tank 3	Tank 1	Tank 2	Tank 3
5 mg	M	6	6	4	10	11	10	9	10	11
	F	6	6	6	10	9	10	10	10	9
60 mg	M	7	6	6	9	10	11	10	11	10
	F	5	6	6	11	10	9	10	9	10

*Artemia nauplii* (Brine Shrimp Direct) were grown at 1.2 g/L for two days (until 24 hours post hatching, instar II-III), in two alternating cultures. Each day the *Artemia* was harvested and, after decanting the cysts and straining the water, the hatched *Artemia* biomass was weighed. The yield was then resuspended to 25 mg/mL and the appropriate volume was dispensed to the fish. For the 60 mg arm, there was approximately two hours between each of the three 20 mg meals. The water flow on the fish tanks was stopped during feeding to prevent flow through. The fish remained on this diet for eight weeks.

The amount of food consumed by the fish was recorded weekly. After a 30-minute feeding period, the fish were transferred to a new tank. The *Artemia* remaining in the tank were filtered through a 40 μm cell strainer and reverse-rinsed into a petri dish. The number of hatched *Artemia* in each dish was then counted under a microscope. For baseline measurements, *Artemia* were dispensed to tanks without fish.

### Body measurements

The physical activity of the fish was recorded in the morning (before feeding) at the start of the diet and at the end of the diet. For this, the fish were transferred, one tank at a time, to a white box with internal dimensions of 13 x 17 cm containing 3.5 L of system water. Following a one-minute acclimatization period, the movement of the fish population was recorded from camera above the box for 30 seconds. Using ImageJ, a threshold was set to select the fish as objects, and watershedding was used to distinguish overlapping objects, the total distance travelled during the recording time was then measured for each tank [26].

The experimental fish were weighed and photographed before and after the dietary intervention in order to determine the weight gained. The fish were anaesthetized, one at a time, in 40 μg/mL tricaine, pH 7, for two minutes or until the heart beat and movement slowed. The fish were then blotted dry and weighed using a fine balance. To determine the length of the fish, each fish was photographed from above next to a ruler and measured using ImageJ [26]. The standard length, from the snout to the base of the tail, was used for calculating the body mass index (BMI): weight (kg) / length (m)^2^.

### RNA sequencing

Gamete samples were collected from fish during the weight gain measurements that followed the dietary intervention. Anaesthetized females were blotted dry, placed in a dish, and pressed gently on the belly to release the eggs. The eggs were gathered with a spatula, transferred to tube containing RA1 buffer (Macherey-Nagel, cat. 740955.250) with β-mercaptoethanol, and stored at -80°C until RNA extraction.

Total RNA was prepared from gamete samples taken from group 3 (dictated by the number of samples obtained from the 5 mg arm). The RNA was filtered with the NucleoSpin RNA kit (Macherey-Nagel, cat. 740955.250) and bound and eluted with columns (17–23,000 nt size range) from the RNA clean and concentrator kit (Zymo cat. no. R1017). The resulting RNA was checked for quality and quantity by Nanodrop, Qubit, and Bioanalyzer.

Library preparation and RNA sequencing was performed by New Zealand Genomics Limited. Total RNA libraries were prepared for the egg samples using the TruSeq stranded total RNA library kit with Ribozero (Illumina). The libraries were run on the HiSeq 2500 (Illumina) to generate single-ended 100 bp reads. The sequence reads were analyzed using the Tuxedo suite [27]. BiNGO, a Cytoscape plugin, was used for gene ontology analysis [28,29]. For comparing diet-induced changes in gene expression with other datasets, the HCOP: Orthology Predictions Search was used to obtain the human orthologs [30]. The data discussed in this publication have been deposited in NCBI’s Gene Expression Omnibus [31] and are available through GEO series accession number GSE81007 (http://www.ncbi.nlm.nih.gov/geo/query/acc.cgi?acc=GSE81007).

### Fertility

The fish used in the dietary intervention were screened to ensure that only those fish capable of breeding (producing at least one egg) with a partner were included in the study. Following the dietary intervention, four spawning experiments were performed, each one week apart. Each spawning experiment involved incrosses of three pairs within each tank and outcrosses of three males and three females to the tank of the other treatment arm (total of 108 pairs for each spawning experiment).

Spawning was induced in the morning with the removal of the barrier between the pairs. A successful breeding event was one where at least one egg was released, as above. The eggs were collected with a sieve, transferred to a dish containing E3 embryo media, and incubated at 28°C. The numbers of fertilized and unfertilized eggs from each breeding pair were counted to calculate the clutch size (total number of eggs) and the fertilization rate. The embryos resulting from each spawning experiment were assessed for viability out to 5 dpf in petri dishes containing 30 larvae.

### The next generation

The fish used in the F_1_ group were derived from the offspring produced by the incrosses performed in the second spawning experiment (in the first spawning experiment there were no breeding pairs in the 5 mg arm). The eggs from multiple pairs across the three F_0_ groups were pooled and separated out into 12 dishes of 27 embryos for each treatment arm (dictated by the number of offspring produced by the 5 mg arm). The F_1_ larvae from both F_0_ arms were then raised under the same standard facility conditions at 7.7 fish/L with offspring mortality and growth (length) measured over two months of maturation.

At two mpf, the fish within each treatment arm were pooled and randomly allocated into two treatment arms, a 5 mg food per fish arm and a 60 mg food per fish arm. This treatment crossover resulted in four F_1_ groups: “5–5” (F_0_: 5 mg, F_1_: 5 mg), “5–60” (F_0_: 5 mg, F_1_: 60 mg), “60–5” (F_0_: 60 mg, F_1_: 5 mg), and “60–60” (F_0_: 60 mg, F_1_: 60 mg). There were three tanks within each treatment arm; these each contained 16 fish that had not been screened for fertility (Table 2). The dietary intervention and measurements were conducted in the same manner as for the F_0_ fish, described above.

**Table 2:** F_1_ group The number of male and female fish used in each F_1_ treatment arm. In total there were 16 fish (4.6 fish/L) in each tank. Mortality prior to the intervention resulted in the loss of one fish from Tank 2 in the 5–5 arm and mortality during the intervention resulted in the loss of five fish from the 5–5 and 5–60 arms (parental 5 mg diet).

Arm	Sex	Tank 1	Tank 2	Tank 3
5–5	M	6	4	4
	F	9	11	10
5–60	M	6	7	8
	F	10	8	7
60–5	M	7	3	2
	F	9	13	14
60–60	M	7	4	6
	F	9	12	10

### Data analysis

Statistical analysis was carried out using *R* statistics [32]. The Student’s unpaired *t*-test was used for the comparison of two means. One-way analysis of variance (ANOVA) with Tukey’s post-hoc comparisons was used for comparing more than two samples. The fertility data was analysed using generalized linear models with clustering at the level of the tank [33]. The chi-squared test was used to determine whether the sex ratio was affected in the F_1_ generation. When determining statistical significance, a *p*-value of ≤ 0.05 was considered significant.

## Results

### Nutrient availability alters energy balance in adult zebrafish

**Fig 1:**
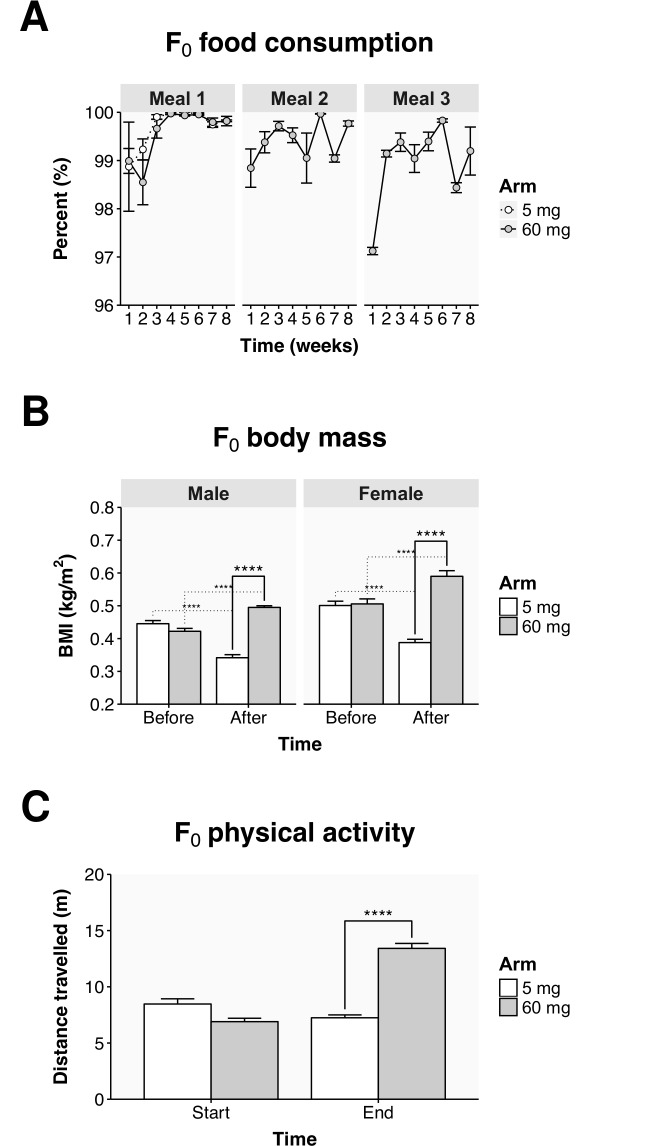
Phenotypic changes resulting from nutrient availability. Gross phenotypic changes are shown only for group 3, for a comparison of the three groups see S1 Fig. (A) Food consumption over the eight weeks of the dietary intervention for the 5 mg arm (meal 1) and the 60 mg arm (meals 1, 2, and 3). The number of *Artemia* remaining following feeding is given as a percentage of the number dispensed to each tank, note the scale begins at 96%. (B) The BMI derived from the weights and lengths of every fish in the 5 and 60 mg treatment arms before and after the dietary intervention. The statistical differences are noted between the treatment arms (solid lines) and within each treatment arm (dotted lines), before and after the diet. (C) The total distance travelled in 30 seconds of swimming for the populations of fish in the 5 and 60 mg treatment arms at the start (week 1) and the end (week 8) of the diet. Values represent the mean ± SEM from the three tanks within each group. Statistically significant differences are noted as **** p ≤ 0.0001.

**Fig 2:**
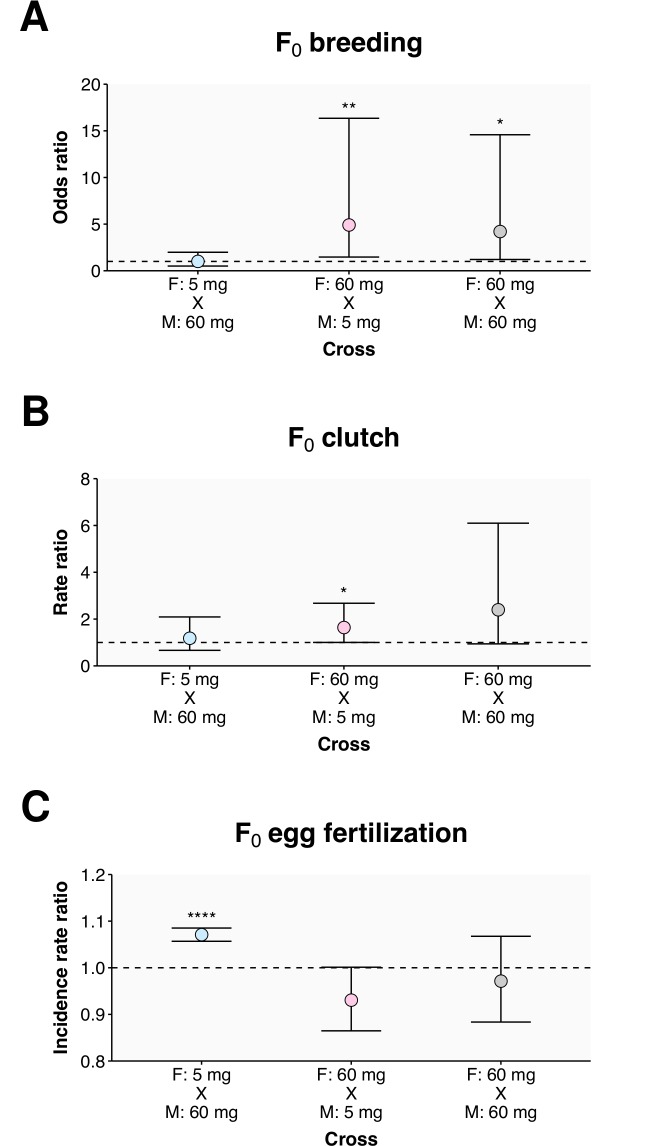
Reproductive consequences of dietary intake. Fertility changes are shown for group 3; for a comparison of the three groups see S2 Fig. The (A) breeding success, (B) clutch size, and (C) fertilization rate is plotted for group 3. Using incrosses within the 5 mg treatment arm (F: 5 mg x M: 5 mg) as the reference for comparison, the effect of the 60 mg treatment was observed using incrosses (F: 60 mg x M: 60 mg) and outcrosses of males (F: 5 mg x M: 60 mg) and females (F: 60 mg x M: 5 mg). The values given represent the odds ratio (A-C), rate ratio (D-F), or incidence rate ratio (G-I) from an average of three pairs from each of three tanks in four spawning experiments. Standard errors were calculated with respect to the tank clusters and are presented as a ± 95% CI. Statistically significant differences are noted as * p ≤ 0.05, ** p ≤ 0.01, and **** p ≤ 0.0001.

We adapted the model of diet-induced obesity in zebrafish [21] using three parallel groups of fish that were given 5 or 60 mg of food each day for eight weeks. The phenotypic changes for group 3 are shown in Figs 1 and 2; while the phenotypic changes for all groups are shown in S1 and S2 Figs.

The fish in both the 5 mg and the 60 mg arm consumed the majority of their food. In the 60 mg arm of group 3, the fish ate, on average, 99.3 ± 0.2% of the *Artemia* they were given (Fig 1A). Counting the *Artemia* remaining after a feeding session suggested that the average ration for each fish was 104 ± 11 *Artemia* in the 5 mg arm and 413 ± 25 *Artemia* (1239 a day) in the 60 mg arm. Only minor consumption differences were observed between the groups (S1A–S1C Fig). In week 2, the fish in the 60 mg arm of group 1 consumed 4.6% (95% CI: 3.7–5.6, *p* < 0.0001) less of their first meal than the fish in the 5 mg arm (S1A Fig). In the last meal of the day, the fish in the 60 mg arm of groups 1 and 3 consumed 1.5% (95% CI: 0.5–2.5, *p* = 0.0017) and 0.5% (95% CI: 0.2–0.7, *p* = 0.0005) less food than in their second meal, respectively (Fig 1A and S1A Fig).

Following the dietary intervention, the physical appearance of the fish differed markedly between the two treatment arms. The treatment arms had a very similar BMI before the diet but after the intervention the BMI of the fish in the 60 mg arm was 1.5 fold greater than the fish in the 5 mg arm (Fig 1B). While the weight of the fish increased relative to the length, it should also be noted that the fish in the 60 mg arm were longer in length after the diet (S1 Table). For example, in group 3, the average standard fish length before the diet was 22.5 mm, but following the diet the lengths of fish in the 60 mg arm had increased 3.2 mm (95% CI: 1.6–4.7, *p* = 0.0007) while the lengths of fish in the 5 mg arm had not changed significantly. Fish in the 60 mg arm also appeared more colorful, with increased sexual divergence in their coloration (S1 Table). This difference was most apparent in group 1, where the sum of the RGB (red, green, blue) values differed by 7% (95% CI: 4–10, *p* = 0.0078) between the males and females in the 60 mg arm but only non-significantly by 2% in the 5 mg arm.

Weight loss in the 5 mg arm indicated that these fish could not represent a normal-fed control, as described previously by Oka et al. [21], and instead represented conditions of dietary restriction. In group 3, the BMI in the 60 mg arm increased 0.07 kg/m^2^ (95% CI: 0.04–0.11, *p* < 0.0001) for males and 0.08 kg/m^2^ (95% CI: 0.05–0.12, *p* < 0.0001) for females (Fig 1B). In contrast, in the 5 mg arm the BMI decreased by 0.10 kg/m^2^ (95% CI: 0.07–0.14, *p* < 0.0001) for males and 0.11 kg/m^2^ (95% CI: 0.08–0.15, *p* < 0.0001) for females (Fig 1B). This decrease in the BMI reflected a drop in the actual weight of the fish in the 5 mg arm (S1 Table). In the 60 mg arm, the males gained 123 mg (95% CI: 81–0.165, *p* < 0.0001) and the females gained 120 mg (95% CI: 74–165, *p* < 0.0001). In the 5 mg arm, the males lost 61 mg (95% CI: 19–103, *p* = 0.007) and the females lost 63 mg (95% CI: 18–109, *p* = 0.008). For reference, the males in group 3 weighed 220 mg and the females weighed 245 mg before the diet. The diets thus reflect two ends of a spectrum of nutrient availability, from abundance to scarcity.

We were interested to know whether the increased food intake would make the fish ‘lazy’ or give them ‘fuel to burn’. Recording the distance travelled by the population of fish in each tank revealed higher levels of swimming activity in the 60 mg arm (Fig 1G–1I and S5 Fig). This effect can be illustrated by group 3 where at the end of the diet the fish in the 60 mg arm travelled a collective distance of 13.4 ± 0.4 m (*p* < 0.0001) while the fish in the 5 mg arm only moved 7.2 ± 0.3 m (Fig 1C). For reference, at the start of the diet the fish in the 60 mg arm travelled 6.9 ± 0.3 m while the fish in the 5 mg arm travelled 8.5 ± 0.5 m (Fig 1C). The data support the idea that there is a correlation in the fish populations between physical activity and energy availability.

### Parental nutrition has reproductive consequences

The fertility of the three groups of experimental fish was assessed following the dietary intervention. Crossing the fish with single partners within each tank revealed that pairs of fish in the 60 mg arm treatment arm were more likely to breed successfully (produce eggs) than the incrosses of fish within the 5 mg arm (Fig 2A). The effect was most pronounced in group 2 (S2B Fig). where a cross of a male and a female from the 60 mg arm (F: 60 mg x M: 60 mg) was 6.2 fold (95% CI: 2.2–17.1, *p* = 0.0004) more likely to be successful than a cross of a male and a female from the 5 mg arm.

As a criterion for their inclusion in the study, all of the experimental fish had bred successfully. However, in the first week following the intervention, none of the individuals within the 5 mg arm were successful in breeding when incrossed. In the three subsequent weeks of fertility analysis, the incrosses within the 5 mg arm did have breeding success, allowing comparisons to be made to other crosses. We found that the odds of breeding increased 4.9 fold (95% CI: 1.5–16.3, *p* = 0.01), compared to incrosses (F: 5 mg x M: 5 mg), when a female from the 60 mg arm was used (F: 60 mg x M: 5 mg) while the inverse cross had no effect (Fig 2A). While this pattern held true for group 2 (S2B Fig), it did not hold for group 1 (S2A Fig), the oldest group, where the breeding success for the F: 5 mg x M: 60 mg cross was significantly greater than that observed in group 2 (*p* < 0.0001) and group 3 (*p* = 0.03). Overall, the results suggest that the female diet contributed more than the male diet towards breeding success.

The dietary intervention had a similar effect on the size of the clutches of eggs produced by the fish (Fig 2B). First, it should be noted that, compared to group 1, the younger fish in group 3 produced smaller clutches; for incrosses in the 5 mg arm this rate was 0.8 fold (95% CI: 0.6–1.0, *p* = 0.03) lower. As with breeding success (above), the clutch sizes were unchanged when males from the 60 mg arm were crossed to females from the 5 mg arm (F: 5 mg x M: 60 mg), relative to incrosses within the 5 mg arm. However, when females from the 60 mg arm were crossed to males within the 5 mg arm (F: 60 mg x M: 5 mg), in group 3 the eggs were produced at a rate 1.6 fold (95% CI: 1.0–2.7, *p* = 0.049) higher than incrosses within the 5 mg arm (Fig 2B). This result is consistent with the observation that the females in the 60 mg arm of group 3 showed a non-significant trend towards having larger ovaries, these were 47 ± 14 mg compared to 21 ± 3 mg in the 5 mg arm (S1 Table). In summary, following the dietary intervention, females in the 60 mg arm were more likely to breed and produce larger clutches of eggs than the females in the 5 mg arm.

In contrast to the trend of increased breeding success and clutch size with increased nutrition, the incidence of egg fertilization did not differ between incrosses within the 60 mg arm and incrosses within the 5 mg arm (S2G–S2I Fig). However, there was a high incidence of egg fertilization in the crosses of males from the 60 mg arm with females from the 5 mg arm (F: 5 mg x M: 60 mg) and a low incidence of egg fertilization in the crosses of males from the 5 mg arm with females from the 60 mg arm (F: 60 mg x M: 5 mg), that was consistent across the three groups (S2G–S2I Fig). In group 3, this difference reached statistical significance with the males from the 60 mg arm increasing the incidence of egg fertilization by 7% (IRR = 1.07, 95% CI: 1.06–1.09, *p* < 0.0001), relative to the incross within the 5 mg arm (Fig 2C). When the different groups were compared, it was observed that the fertilization rate with the 60 mg males (F: 5 mg x M: 60 mg) was 4% (IRR = 1.04, 95%CI: 1.01–1.06, *p* = 0.005) higher for the young group 3, compared to group1. Together, the data demonstrate that the 60 mg diet increased the breeding success and clutch sizes for females and increased fertilization rates for males. For the raw percent breeding, clutch size, and egg fertilization data see S3 Fig.

### The transcriptome of eggs varies with diet

We wanted to know whether the relative abundances of egg RNA transcripts varied with diet. To this end we collected egg samples from three females from both treatment arms of group 3. From these samples we obtained high yield (average: 208 ng/μL), high quality (average RIN: 9.9), RNA from the unfertilized eggs of the experimental fish. Following sequencing we received, on average, 25.3 million sequencing reads for each egg sample, 98.2% of which had a Q score greater than or equal to 30. Alignment resulted in an average of 82.1% of the reads being mapped to the genome (Zv9). The FPKM values for these reads were found to be similarly distributed between the samples and treatment arms (S6A Fig and S6B Fig). As such, there did not appear to be any large scale differences in sequencing quality between the treatment arms that might hinder the ability to make comparisons.

**Fig 3:**
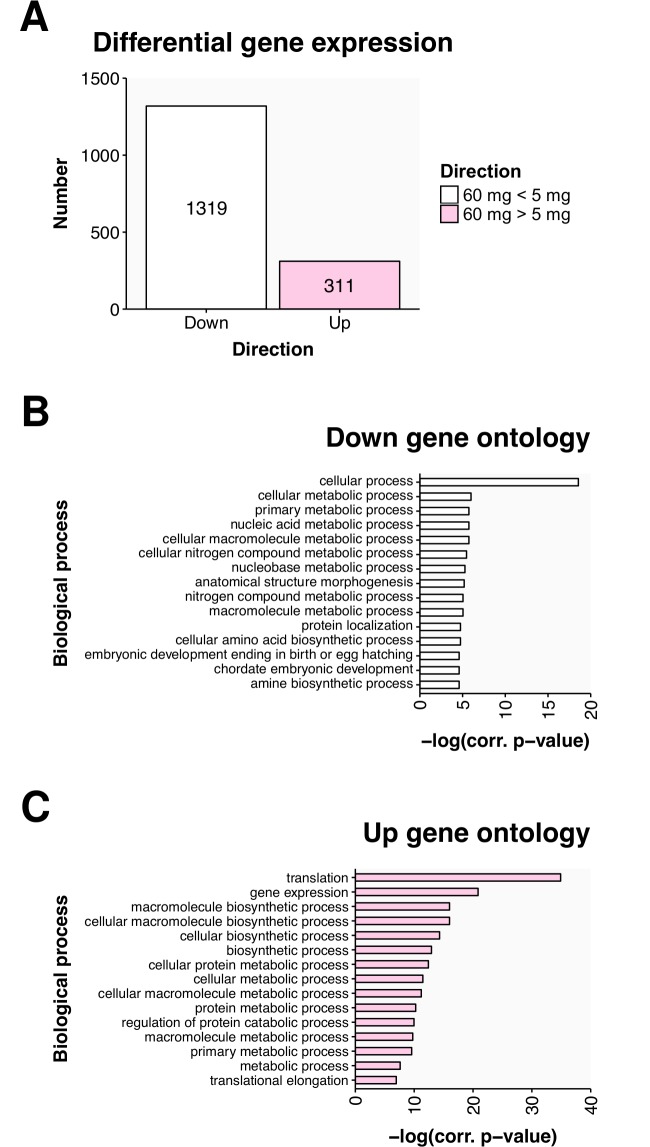
RNA sequencing revealed diet-induced differences in egg transcript deposition. (A) The differentially expressed genes are shown in terms of the number of genes significantly altered in each direction. The significance cut-off was–log(*p* = 0.005) = 2.3. The top 15 biological processes overrepresented within the list of genes significantly (B) downregulated and (C) upregulated in the 60 mg arm are given as bars with the size indicating the significance of the enrichment.

Analysis of the differentially expressed genes revealed diet-induced changes in transcript complement within the eggs. After correcting for multiple tests, there were 1630 transcripts that were found to be differentially abundant between the two treatment arms. Interestingly, there were 1319 transcripts that were under-represented in the eggs from the females in the 60 mg arm and only 311 transcripts that were enriched, relative to the eggs from the females of the 5 mg arm (Fig 3A). Gene ontology revealed that among the transcripts downregulated by the 60 mg diet (upregulated by the 5 mg diet), those involved in nucleic acid metabolism (n = 67, corrected *p* < 0.003), nitrogen metabolism (n = 83, corrected *p* < 0.004), and morphogenesis (n = 48, corrected *p* < 0.006) were overrepresented (Fig 3B, S3 Table). Among the transcripts that were upregulated by the 60 mg diet (downregulated by the 5 mg diet), those involved in translation (n = 25, corrected *p* < 0.0001), gene expression (n = 37, corrected *p* < 0.0001), and biosynthesis (n = 37, corrected *p* < 0.0001) were overrepresented (Fig 3C, S4 Table). Hence, we observed a diet-induced shift in the deposition of egg transcripts.

The gene expression changes observed in our study showed similarities to those found in other studies. We found four studies on the Gene Expression Omnibus that had examined the impact of dietary intake on the transcriptome of the female gonad; GSE69716 (monkey), GSE76232 (sheep), GSE76002 (rat), and GSE7502 (mouse). GO categories that significantly altered in the monkey, sheep, and mouse studies were also altered in our zebrafish study. The common processes were chromatin assembly and DNA packaging (monkey), developmental and metabolic process (sheep), and transcription (mouse).

Genes found in this study to show differential expression in zebrafish eggs with diet were also altered by diet in mouse ovaries (GSE7502). Using the human orthologs as a proxy for the zebrafish and mice genes we found 21 genes (S5 Table) that were upregulated in the ovaries of fish on the 5 mg diet (downregulated in the 60 mg arm) and upregulated in the ovaries of mice following caloric restriction [34]. These genes were found to be involved in digestion (n = 4, corrected *p* < 0.0001) and carbohydrate metabolism (n = 6, corrected *p* = 0.002).

### Embryonic phenotype is affected by parental nutrition

To determine if the parental diet influenced the embryonic phenotype we examined several aspects of the offspring’s development during their first five days of life. Of the eggs that had been fertilized, those from the fish in the 60 mg treatment arm were larger (at 6 hpf) than those produced by the fish in the 5 mg arm (S7A–S7C Fig). This effect was most apparent when comparing the cross of females from the 60 mg arm and males from the 5 mg arm (F: 60 mg x M: 5 mg) with the cross of females from the 5 mg arm and males from the 60 mg arm (F: 5 mg x M: 60 mg). In group 2, the eggs produced by females from the 60 mg arm had a yolk diameter of 675 ± 2 μm; this was significantly greater (*p* = 0.012) than the yolk diameter from the 5 mg arm (663 ± 2 μm; *p* = 0.012; S7B Fig). We also observed a nonsignificant trend towards larger chorions, which when normalized by the diameter of the yolk, were 4% larger (on average, across the three groups) for the eggs from females in the 60 mg arm compared to the eggs from the females in the 5 mg arm.

The fish of both treatment arms in the three parental groups produced fertilized eggs that could successfully grow into larvae. The fertilized eggs hatched at the expected rate, typically at 2 dpf, with 99% (average across all groups) having hatched by 3 dpf. Early survival, out to 5 dpf, was high at 97% (average across all groups) and not affected by parental diet. Tail malformation in larvae, occurred sporadically at a frequency of 1% (average across all groups) and was not affected by the parental diet. However, there was acephaly (larvae that developed without a head) among 0.5% of offspring produced by incrosses within the 60 mg arm. This malformation was apparent at 1 dpf, in group 1 (n = 1 in 268 embryos), group 2 (n = 2 in 317 embryos), and group 3 (n = 1 in 227 embryos), and was never observed in crosses with the 5 mg arm. In general, however, the offspring of both treatment arms were viable.

We were interested to know whether the parental diet could alter lipid deposition in the larvae. At 5 dpf, the larvae produced by the fish in the 60 mg arm showed increased staining for lipids (S7D–S7F Fig). This effect was significant in the larvae produced by group 2, where the larval staining absorbance was 1.07 ± 0.18 for incrosses within the 60 mg arm; this was larger (*p* = 0.002) than the staining absorbance of 0.63 ± 0.03 noted for incrosses within the 5 mg arm (S7E Fig). The females made a large contribution to amount of lipid in the larvae, with the absorbance also being greater than the incross within the 5 mg arm (*p* = 0.004), at 0.97 ± 0.12, when the females from the 60 mg arm were crossed to the males from the 5 mg arm (F: 60 mg x M: 5 mg). Our data show that the parental diet can alter the phenotype of the offspring even at a very early stage of development.

### Parental diet affects F1 survival and energy expenditure

**Fig 4:**
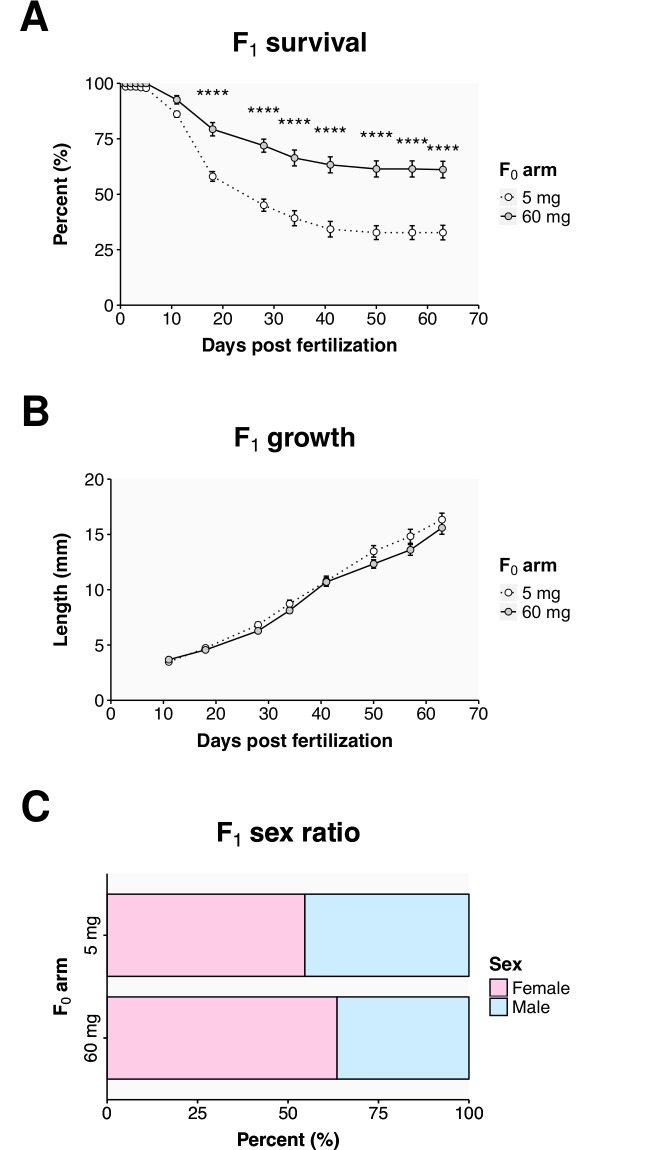
Parental diet affects F_1_ survival. (A) The survival of pooled offspring from incrosses within the 5 and 60 mg treatment arms over their maturation. (B) The growth of the offspring as determined by the length of 5 randomly selected fish from each tank from each parental treatment arm (n = 60 per condition) over the course of their development. (C) The sex ratio of the offspring. Values represent the mean ± SEM from three tanks within each group. Statistically significant differences are noted as * p ≤ 0.05 and **** p ≤ 0.0001.

**Fig 5:**
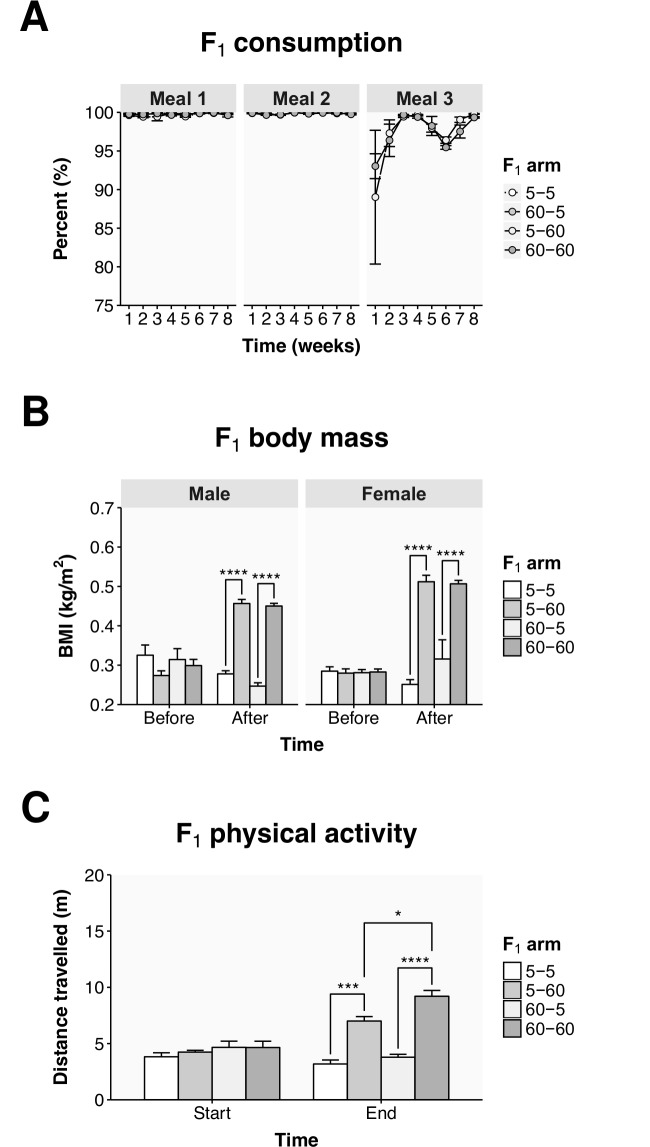
Parental diet affects F_1_ physical activity. (A) Food consumption over the eight weeks of the dietary intervention for the F_1_ 5 mg arms (meal 1) and the F_1_ 60 mg arms (meals 1, 2, and 3). The number of *Artemia* remaining following feeding is given as a percentage of the number dispensed to each tank, note the scale begins at 75%. (B) The BMI derived from the weights and lengths of every fish in the 5 and 60 mg treatment arms before and after the dietary intervention. (C) The total distance travelled in 30 seconds of swimming for the populations of fish in the 5 and 60 mg treatment arms at the start (week 1) and the end (week 8) of the diet. Values represent the mean ± SEM from the three tanks within each group. Statistically significant differences are noted as * p ≤ 0.05 and **** p ≤ 0.0001.

We were interested to know how the parental diet would affect the long-term health of the offspring. To this end we raised an F_1_ group from a pool of offspring that was produced by incrosses within the treatment arms of the three parental groups. The survival and growth of this F_1_ group was tracked through juvenile development (Fig 4A and 4B). While survival out to 5 dpf was not affected by the parental diet (above), a difference in survival did became apparent at 10 dpf (Fig 4A). By 18 dpf, the survival in the progeny of the 5 mg arm was significantly lower (*p* < 0.0001) at 58 ± 2%, than the survival in the progeny of the 60 mg arm (79 ± 3%). At the end of the measurement period, at 63 dpf, the survival in the progeny of the 5 mg arm was 28% lower (95% CI: 16–41, *p* < 0.0001) than the survival in the progeny of the 60 mg arm. The parental diet did not, however, result in any significant differences in the growth of the progeny, in terms of their body length over time (Fig 4B). The resulting progeny of the 60 mg arm did show a skew towards females in the progeny, but this difference was not (*p* = 0.2) significant (Fig 5C). Therefore, the F_1_ generation represented a group of fish that differed according to their parental nutritional environment and that showed altered survival into adulthood.

To find out how nutrient availability in the parental generation might affect the response to nutrient availability in the offspring we conducted an intergenerational crossover study. The F_1_ progeny, from both the 5 mg arm and the 60 mg arm, were again given either 5 or 60 mg of food each day, resulting in four F_1_ treatment arms. The F_1_ fish consumed the majority of their food with the fish given 60 mg each day (5–60 and 60–60) eating, on average, 98 ± 0.4% of the *Artemia* they were given (Fig 5A). In the last meal of the day, the fish given 60 mg each day consumed 2.5% (95% CI: 1.5–3.5, *p* < 0.0001) less food than their second meal. It was noticed that at the start of the diet, in week 1, the fish from the 5 mg parental arm (5–60) ate 4% less of their third meal than the fish from the 60 mg parental arm (60–60), but this was not significant.

We wanted to know how the parental diet would influence weight gain in the offspring. The arms in the F_1_ group did not show a difference in BMI before the dietary intervention (Fig 5B). After the diet though, the fish that were in the 60 mg arm (5–60 and 60–60) had a higher BMI than the fish that were in the 5 mg arm (5–5 and 60–5). While the males in the 5 mg arm that had parents in the 60 mg arm (60–5) showed a slightly lower BMI than the males in the 5 mg that had parents in the 5 mg arm (5–5), this was not significant. As such, the parental treatment arms did not affect the BMI of the fish in the F_1_ generation.

Interestingly, an intergenerational effect was observed in swimming activity (Fig 5C). At the start of the diet, those fish in the 60 mg arm (5–60 and 60–60) were travelling a slightly larger distance than those fish in the 5 mg arm (5–5 and 60–5), but this was not significant. As in the F_0_ parental generation, at the end of the diet, those fish in the 60 mg arm showed increased swimming activity. However, of note is the difference between the 5–60 and 60–60 arms. At the end of the diet those fish in the 5–60 arm collectively travelled 7.0 ± 0.4 m, while those fish in the 60–60 arm travelled 9.2 ± 0.5 m (*p* = 0.029). The high dietary intake in the parental generation therefore appeared to allow for increased physical activity in the subsequent generation when exposed again to a high dietary intake.

## Discussion

The zebrafish represents a tractable model to investigate metabolic diseases in adulthood. It is therefore a concern that dietary manipulations used in one lab may not be readily reproduced by another. When we adapted the Oka et al. feeding protocol we observed significant weight loss on the ‘control’ diet. However, the fish receiving the control diet in the study by Oka et al. did not show weight loss; rather, there was a small weight gain [21]. This suggests that different fish populations may respond differently to dietary regimes depending on their original facility conditions.

A combination of factors may underlie the different response to the Oka et al. feeding protocol that we observed in this study. When considering the control diet, Oka et al. aimed to provide the fish with 20 calories [21], citing Pannevis et al. [35] who estimated the maintenance energy required by a 300 mg *Danio*. While the fish used in our experiment were older, they were still under 300 mg and so the caloric requirement should have been equivalent. Furthermore, weight loss in the control diet was still observed in the F_1_ fish, which were young and small at the start of the intervention. Another difference is that Oka et al. used freshly hatched *Artemia* while this study used 1-day post-hatch *Artemia*; this could have resulted in 22–37% less energy for the fish in this study [36]. In addition to these factors we expect a contribution from the background levels of feeding in the facility. This study highlights a need to standardize the zebrafish feeding protocols [37] and conduct more basic research on zebrafish nutrition and metabolism.

Organisms must make important decisions about how to invest their limited resources among competing processes such as growth and reproduction [38]. The treatment arms used in this study reflect a spectrum of nutrient availability, from restriction in the 5 mg arm to excess in the 60 mg arm. We observed a correlation between the availability of metabolic resources and levels of physical activity; this is consistent with the finding that chronic food-deprivation in zebrafish reduces levels of physical activity [39]. We expect that the size of the fish, which was altered by the diet, contributed to activity levels. Investing energy in swimming activity would provide several advantages for a fish in terms of escaping predators, searching for food, and finding a mate [40].

Increased resource availability improved breeding success and egg production for females and improved fertilization rates for males. Given the important role of male behavior in the mating process, breeding success is not likely to be explained solely by the levels of physical activity [41]. In contrast to breeding success, the clutch size is a more specific phenotype and might be linked directly to the metabolic resources available to the females. Unlike mammalian females that produce a limited number of germ cells that are arrested in meiosis prior to birth, adult fish ovaries contain mitotic germ cells and are always producing new eggs [42]. While the increased resource availability improved the fertilization rates from males, likely due to improved sperm quality, there were decreased fertilization rates from the females. Given increased clutch sizes, lower fertilization rates might allow the best eggs to be selected for further development.

A biological association between obesity and infertility is well described in humans, for both males [43] and females [44]. However, in our study, the fish did not appear to develop obesity-related infertility. Zebrafish may not recapitulate the human obesity condition due to species-specific differences in sedentary behavior or differences in diet composition such the amount of fat or sugar consumed [45]. It is possible that establishing the amount of food required to reach satiety and optimizing the duration of the dietary intervention could lead to conditions that better reproduce human obesity in fish populations. Furthermore, while weight gain can be detrimental to modern human health, under environmental conditions where nutrients are limited, gaining weight can be advantageous [46].

The parental diet influenced the development of the offspring and altered their energy balance in adulthood. As highly fecund animals, zebrafish produce many offspring that have a low probability of survival through maturation [47]. We found that nutrient availability before breeding improved offspring quality, as evident by the increased probability of surviving to adulthood. The parental diet also influenced the regulation of energy utilization as indicated by the increased levels of activity when both the F_0_ and the F_1_ received the high abundance diet. One possible explanation is that parental exposure to a scarce food supply resulted in the offspring being metabolically programmed towards energy conservation [3]. This poor parental environment might then, under certain environmental conditions, provide a fitness advantage for the offspring.

Transcriptional profiling of the unfertilized eggs showed signs of adaptation towards greater resource availability. Comparing the changes to those observed in other studies highlighted the link between diet, metabolism, and transcription. In our study, high nutrient availability led to an enrichment of factors promoting growth in the eggs, a finding supported by the increased yolk size and lipid content in the embryos. The nutrient-restricted eggs showed a relative upregulation of many genes suggesting a need to activate more pathways in preparation for their development. Our data point to the process of translation as a contributor to the intergenerational effects of parental diet. This finding is supported by recent studies in animal models that have shown that tRNA-derived small RNAs [12] and ribosomal RNA copy number [48] can be affected by parental and be transmitted to the next generation.

The availability of nutrients influenced the allocation of metabolic resources in the embryo. High nutrient availability led to enrichment of growth promotion pathways, better survival, and higher motility in the offspring. This result suggests that these individuals had inherited more resources, promoting their fitness. In contrast, low nutrient availability led to lower survival, conservation of motile energy and relative upregulation of metabolic pathways, suggesting embryos had to conserve and build metabolic resources, having not inherited these from their parents. In this way, parental nutritional status can alter metabolic decision-making in offspring with consequent impact on survival and energy expenditure. Conservation of these phenomena through the animal kingdom remains to be determined.

## Supporting Information

S1 Fig Phenotypic changes resulting from nutrient availability. Gross phenotypic changes in all three of the groups used in the study. (A-C) Food consumption over the eight weeks of the dietary intervention for the 5 mg arm (meal 1) and the 60 mg arm (meals 1, 2, and 3). The number of *Artemia* remaining following feeding is given as a percentage of the number dispensed to each tank, note the scale begins at 85%. (D-F) The BMI derived from the weights and lengths of every fish in the 5 and 60 mg treatment arms before and after the dietary intervention. The statistical differences are noted between the treatment arms (solid lines) and within each treatment arm (dotted lines), before and after the diet. (G-I) The total distance travelled in 30 seconds of swimming for the populations of fish in the 5 and 60 mg treatment arms at the start (week 1) and the end (week 8) of the diet. Values represent the mean ± SEM from three tanks within each group. Statistically significant differences are noted as * p ≤ 0.05, ** p ≤ 0.01, *** p ≤ 0.001, and **** p ≤ 0.0001.
(TIF)

S2 Fig Reproductive consequences of dietary intake. Fertility changes in all three of the groups used in the study. The breeding success (A-C), clutch size (D-F), and fertilization rate (G-I) is given for each group. Using incrosses within the 5 mg treatment arm (F: 5 mg x M: 5 mg) as the reference for comparison, the effect of the 60 mg treatment was observed using incrosses (F: 60 mg x M: 60 mg) and outcrosses of males (F: 5 mg x M: 60 mg) and females (F: 60 mg x M: 5 mg). The values given represent the odds ratio (A-C), rate ratio (D-F), or incidence rate ratio (G-I) from an average of three pairs from each of three tanks in four spawning experiments. Standard errors were calculated with respect to the tank clusters and are presented as a ± 95% CI. Statistically significant differences are noted as * p ≤ 0.05, ** p ≤ 0.01, *** p ≤ 0.001, and **** p ≤ 0.0001.
(TIF)

S3 Fig Raw fertility changes with dietary intake. Fertility changes in all three of the groups used in the study. (A-C) The breeding success is percent of pairs that bred out of those set up. (D-F) The clutch size is the number of eggs produced by a pair that was successful in breeding. (G-I) The fertilization rate is the percent of eggs that had started to develop by 6 hpf out of the total number of eggs counted. Using incrosses within the 5 mg treatment arm (F: 5 mg x M: 5 mg) as the reference for comparison, the effect of the 60 mg treatment was observed using incrosses (F: 60 mg x M: 60 mg) and outcrosses of males (F: 5 mg x M: 60 mg) and females (F: 60 mg x M: 5 mg). Values represent the mean ± SEM for the three tanks in four spawning experiments, for statistical analysis see S2 Fig.
(TIF)

S4 Fig Adult lipid. (B) Representative images of Oil Red O stained sections taken from the indicated region (A, line) during the F0 necropsy. The total ORO area (S1 Table) was determined by setting a color threshold (B, bottom row) based on image hue, saturation, and brightness. The scale bar given is 1 mm and is relevant for all the sections shown.
(TIF)

S5 Fig Swimming activity. The swimming paths of the fish following the dietary is shown for the 5 and 60 mg treatment arms from each group. The distance travelled by each fish was recorded over 30 seconds and each panel shows the tank of fish that had the average swimming distance for the condition it represents.
(TIF)

S6 Fig RNA sequencing reads. The distribution of mapped read numbers is shown (A) as a density plot for the 5 and 60 mg conditions and (B) as a box plot for the replicate samples. FPKM: fragments per kilobase of transcript per million fragments mapped. (C) The differentially expressed genes are shown in terms of the the fold change and significance in each direction. The significance cut-off was–log(*p* = 0.005) = 2.3.
(TIF)

S7 Fig Parental diet influences embryo size and lipid composition. (A-C) The diameter of the egg yolk for the fertilized eggs produced from incrosses within the 5 and 60 mg treatment arm and the outcrosses between them. Values represent the mean ± SEM for 10 individual eggs from each cross from three tanks within each group over four spawning experiments. (D-F) The amount of lipid present at 5 dpf, measured by Oil Red O absorbance at 490 nm. Values represent the mean ± SEM for pools of 30 larvae from each cross from three tanks within each group over three spawning experiments. Statistically significant differences are noted as * p ≤ 0.05 and ** p ≤ 0.01.
(TIF)

S1 File Supporting information. (DOC)

S1 Table Phenotypic changes resulting from nutrient availability. Body parameters describing the F0 fish in groups 1, 2, and 3. Standard length, caudal fin length, total RGB, and weight were measured for every fish and the values are given as the mean ± SEM for the three tanks. Gonad and brain weight were measured for two males and two females from each tank and the values are given as the mean ± SEM for the three tanks. Less fish from cohort 1 were available for dissection so this data is absent. Lipid measurements were made from three males and three females from each tank and the values are given as the mean ± SEM for the three tanks.
(XLSX)

S2 Table Differentially expressed genes in the eggs. The 1,630 genes found by RNA-seq to have significantly different expression in the eggs of the 5 and 60 mg females of group 3. Each gene is identified by the gene_id, gene_symbol, and chr_locus. The expression of each gene in the 5 mg and 60 mg samples is given as the FPKM in value_1 and value_2, respectively. The direction and degree of the differential expression is given as the log2(fold_change). The statistical significance of the difference is indicated by the test_stat, p_value, and q_value. Only the significantly altered genes are provided, the significance cut-off was *p* = 0.005.
(XLSX)

S3 Table Biological processes downregulated in eggs by the 60 mg diet. Transcripts significantly downregulated by the 60 mg diet (upregulated by the 5 mg diet) were analysed for gene ontology using BiNGO, a Cytoscape plugin [1,2]. Provided in the table is the GO-ID, a description of the biological process, the statistical significance, the number of genes included in the category, and the gene names.
(XLSX)

S4 Table Biological processes upregulated in eggs by the 60 mg diet. Transcripts significantly upregulated by the 60 mg diet (downregulated by the 5 mg diet) were analysed for gene ontology using BiNGO, a Cytoscape plugin [1,2]. Provided in the table is the GO-ID, a description of the biological process, the statistical significance, the number of genes included in the category, and the gene names.
(XLSX)

S5 Table Genes showing differential expression in zebrafish and mice ovaries. The list of 21 human orthologs that were significantly upregulated by the 5 mg diet (downregulated by the 60 mg diet) in this study and also found to be upregulated by caloric restriction in the mouse ovary by Sharov et al. [3].
(XLSX)
