# Supplementary figures and images for: Dietary Intake Influences Adult Fertility and Offspring Fitness in Zebrafish
Source: PLoS One. 2016 Nov 21;11(11):e0166394. doi: 10.1371/journal.pone.0166394 (PMC5117665; doi:10.1371/journal.pone.0166394)

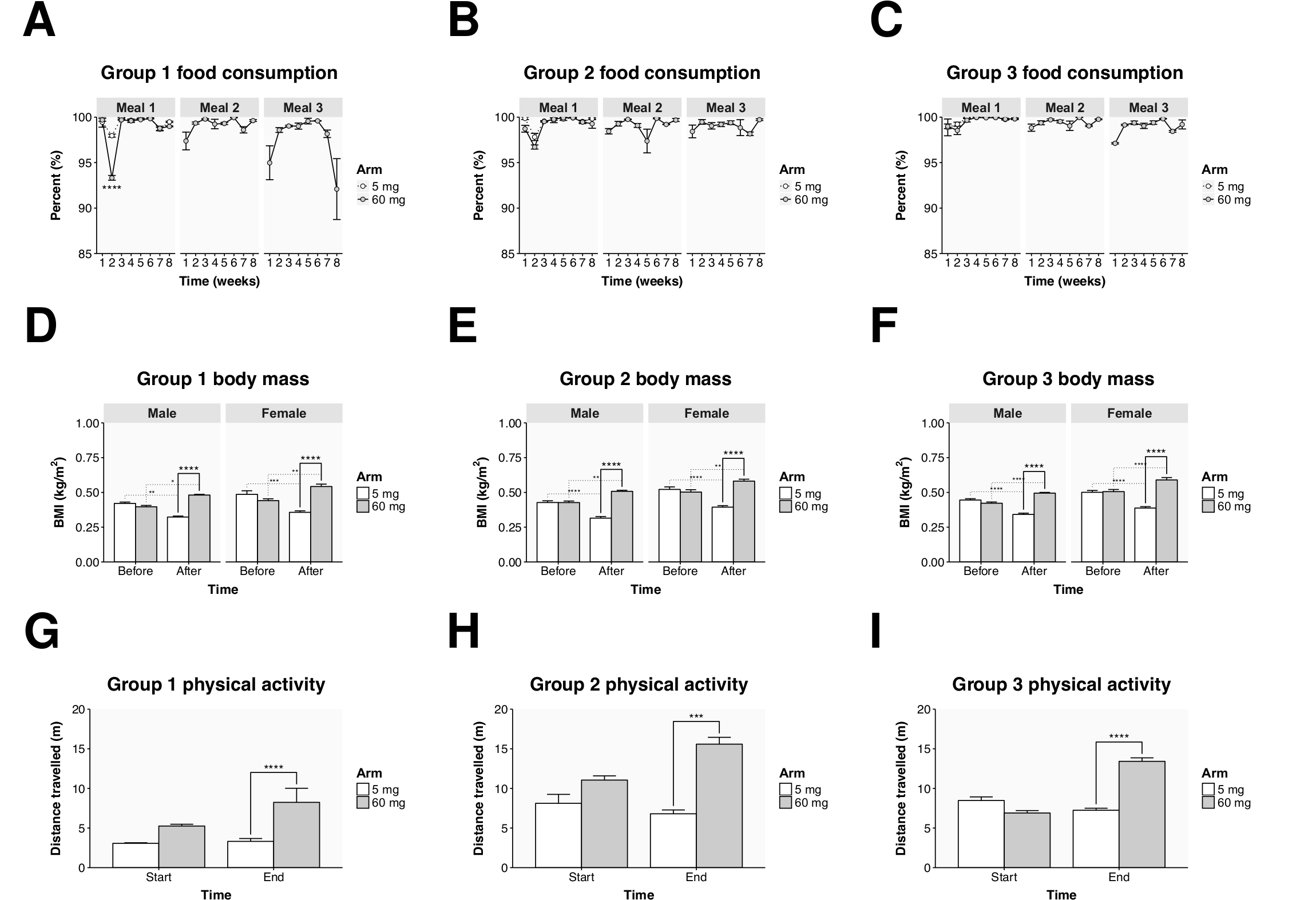

Supplement: S1 Fig — Gross phenotypic changes in all three of the groups used in the study. (A-C) Food consumption over the eight weeks of the dietary intervention for the 5 mg arm (meal 1) and the 60 mg arm (meals 1, 2, and 3). The number of Artemia remaining following feeding is given as a percentage of the number dispensed to each tank, note the scale begins at 85%. (D-F) The BMI derived from the weights and lengths of every fish in the 5 and 60 mg treatment arms before and after the dietary intervention. The statistical differences are noted between the treatment arms (solid lines) and within each treatment arm (dotted lines), before and after the diet. (G-I) The total distance travelled in 30 seconds of swimming for the populations of fish in the 5 and 60 mg treatment arms at the start (week 1) and the end (week 8) of the diet. Values represent the mean ± SEM from three tanks within each group. Statistically significant differences are noted as * p ≤ 0.05, ** p ≤ 0.01, *** p ≤ 0.001, and **** p ≤ 0.0001. (TIF) [file pone.0166394.s001.tif]

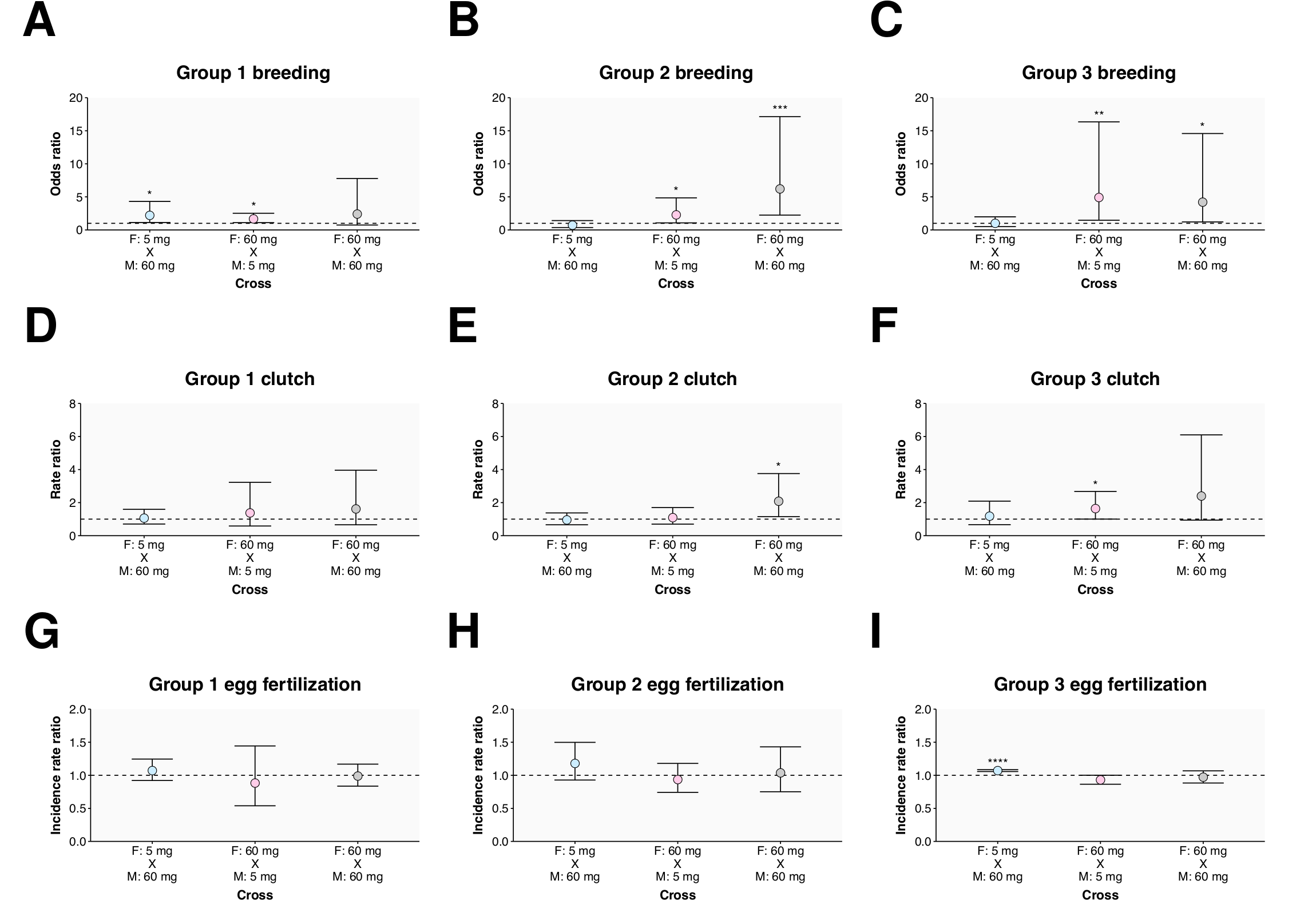

Supplement: S2 Fig — Fertility changes in all three of the groups used in the study. The breeding success (A-C), clutch size (D-F), and fertilization rate (G-I) is given for each group. Using incrosses within the 5 mg treatment arm (F: 5 mg x M: 5 mg) as the reference for comparison, the effect of the 60 mg treatment was observed using incrosses (F: 60 mg x M: 60 mg) and outcrosses of males (F: 5 mg x M: 60 mg) and females (F: 60 mg x M: 5 mg). The values given represent the odds ratio (A-C), rate ratio (D-F), or incidence rate ratio (G-I) from an average of three pairs from each of three tanks in four spawning experiments. Standard errors were calculated with respect to the tank clusters and are presented as a ± 95% CI. Statistically significant differences are noted as * p ≤ 0.05, ** p ≤ 0.01, *** p ≤ 0.001, and **** p ≤ 0.0001. (TIF) [file pone.0166394.s002.tif]

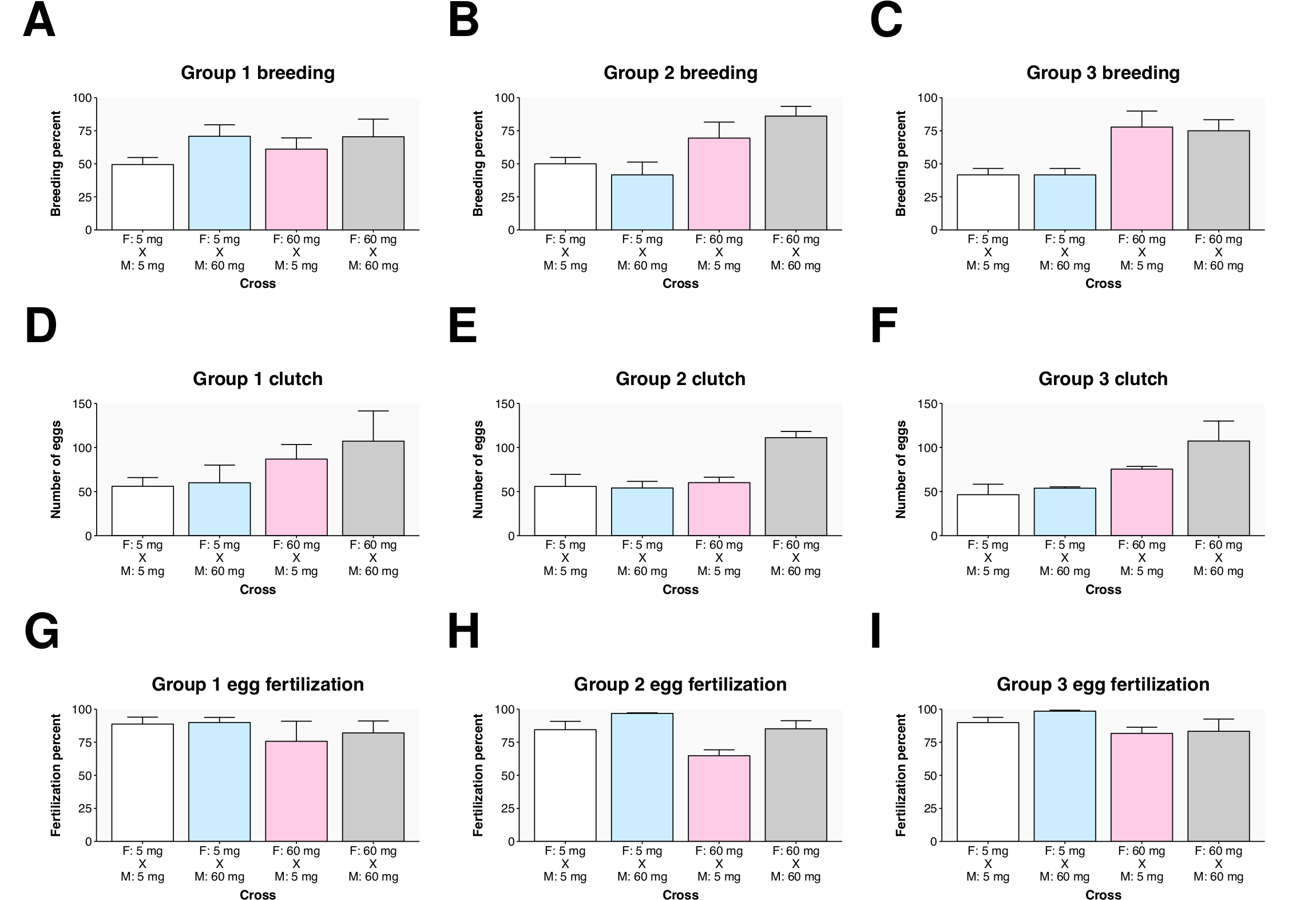

Supplement: S3 Fig — Fertility changes in all three of the groups used in the study. (A-C) The breeding success is percent of pairs that bred out of those set up. (D-F) The clutch size is the number of eggs produced by a pair that was successful in breeding. (G-I) The fertilization rate is the percent of eggs that had started to develop by 6 hpf out of the total number of eggs counted. Using incrosses within the 5 mg treatment arm (F: 5 mg x M: 5 mg) as the reference for comparison, the effect of the 60 mg treatment was observed using incrosses (F: 60 mg x M: 60 mg) and outcrosses of males (F: 5 mg x M: 60 mg) and females (F: 60 mg x M: 5 mg). Values represent the mean ± SEM for the three tanks in four spawning experiments, for statistical analysis see S2 Fig. (TIF) [file pone.0166394.s003.tif]

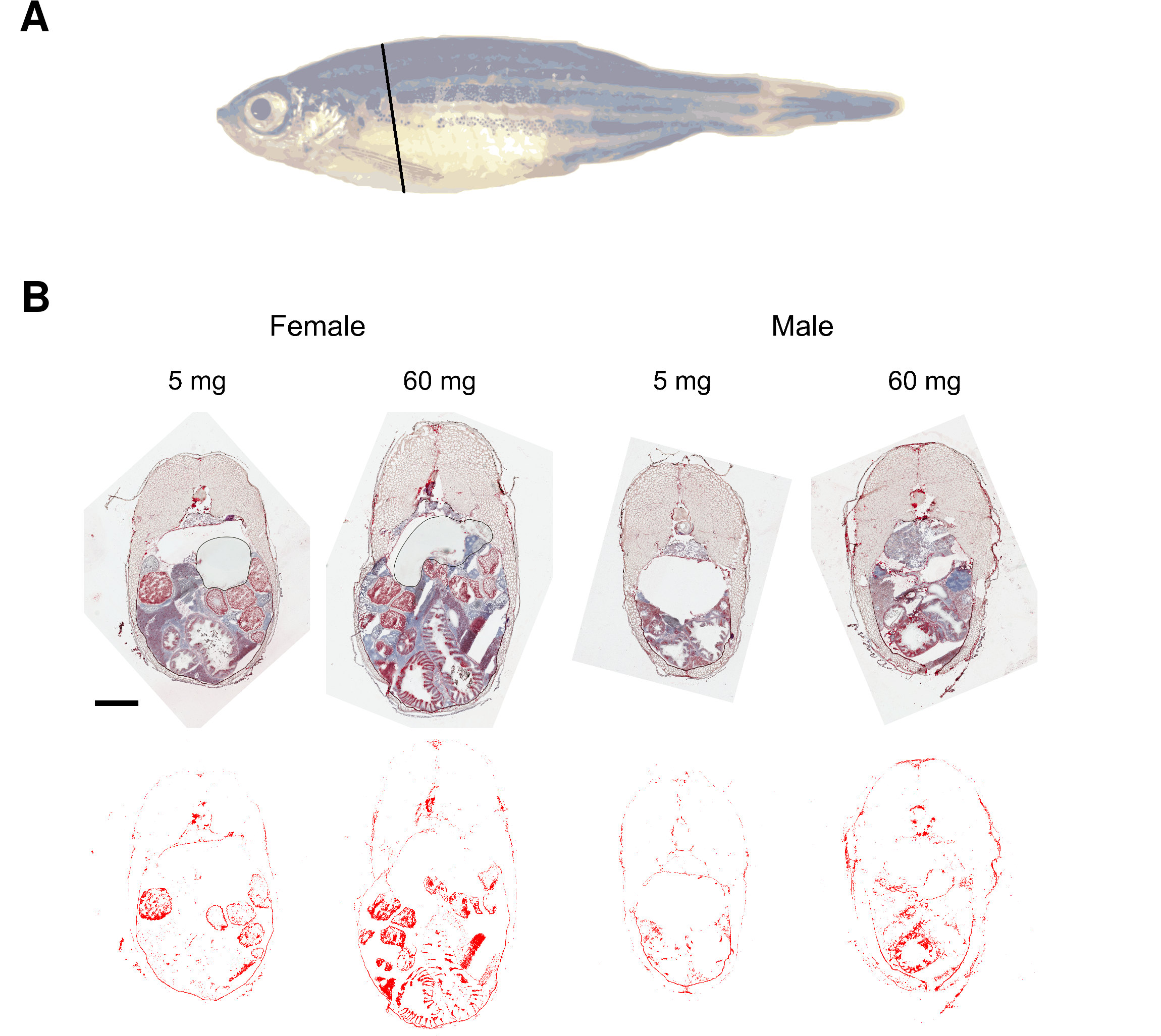

Supplement: S4 Fig — (B) Representative images of Oil Red O stained sections taken from the indicated region (A, line) during the F0 necropsy. The total ORO area (S1 Table) was determined by setting a color threshold (B, bottom row) based on image hue, saturation, and brightness. The scale bar given is 1 mm and is relevant for all the sections shown. (TIF) [file pone.0166394.s004.tif]

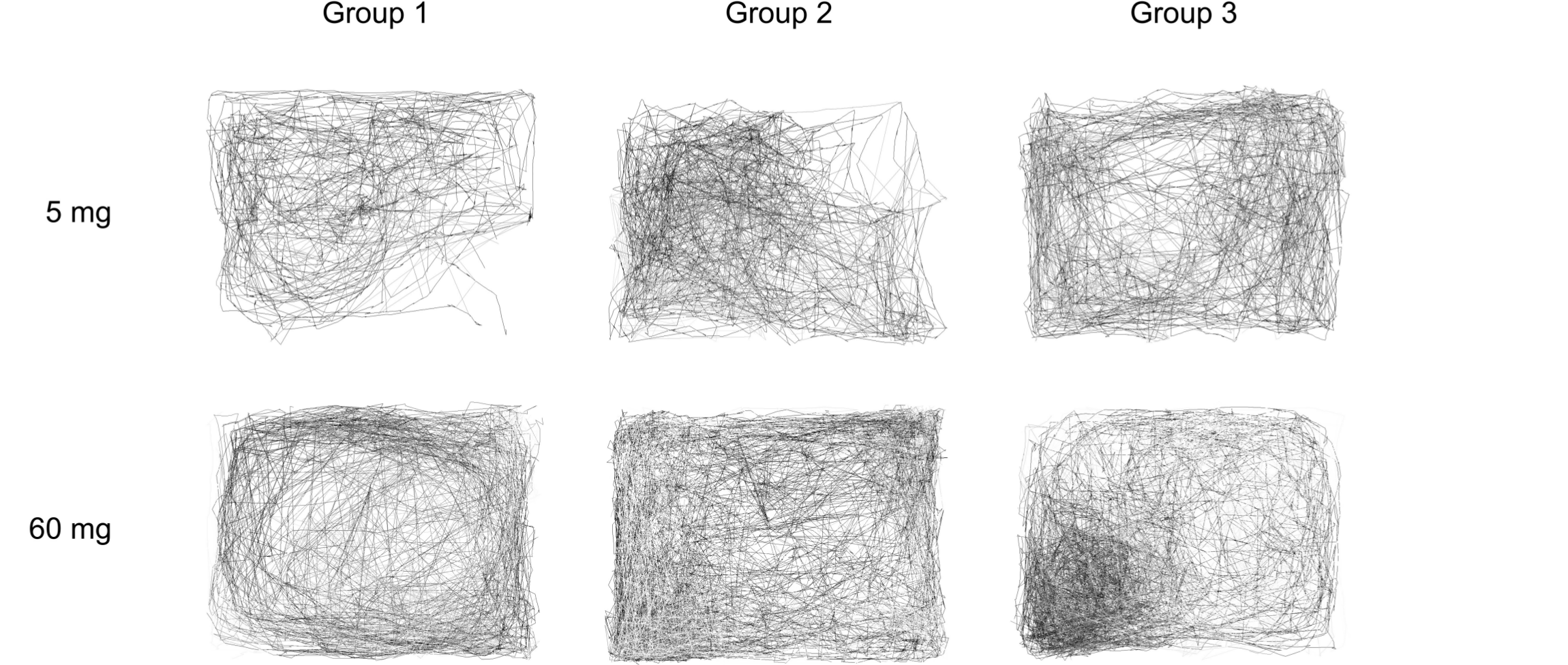

Supplement: S5 Fig — The swimming paths of the fish following the dietary is shown for the 5 and 60 mg treatment arms from each group. The distance travelled by each fish was recorded over 30 seconds and each panel shows the tank of fish that had the average swimming distance for the condition it represents. (TIF) [file pone.0166394.s005.tif]

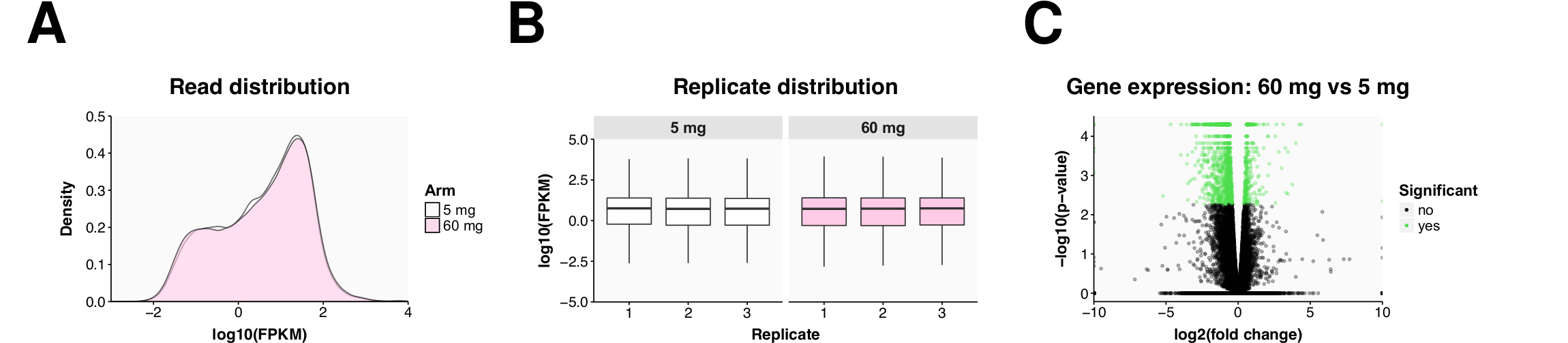

Supplement: S6 Fig — The distribution of mapped read numbers is shown (A) as a density plot for the 5 and 60 mg conditions and (B) as a box plot for the replicate samples. FPKM: fragments per kilobase of transcript per million fragments mapped. (C) The differentially expressed genes are shown in terms of the the fold change and significance in each direction. The significance cut-off was–log(p = 0.005) = 2.3. (TIF) [file pone.0166394.s006.tif]

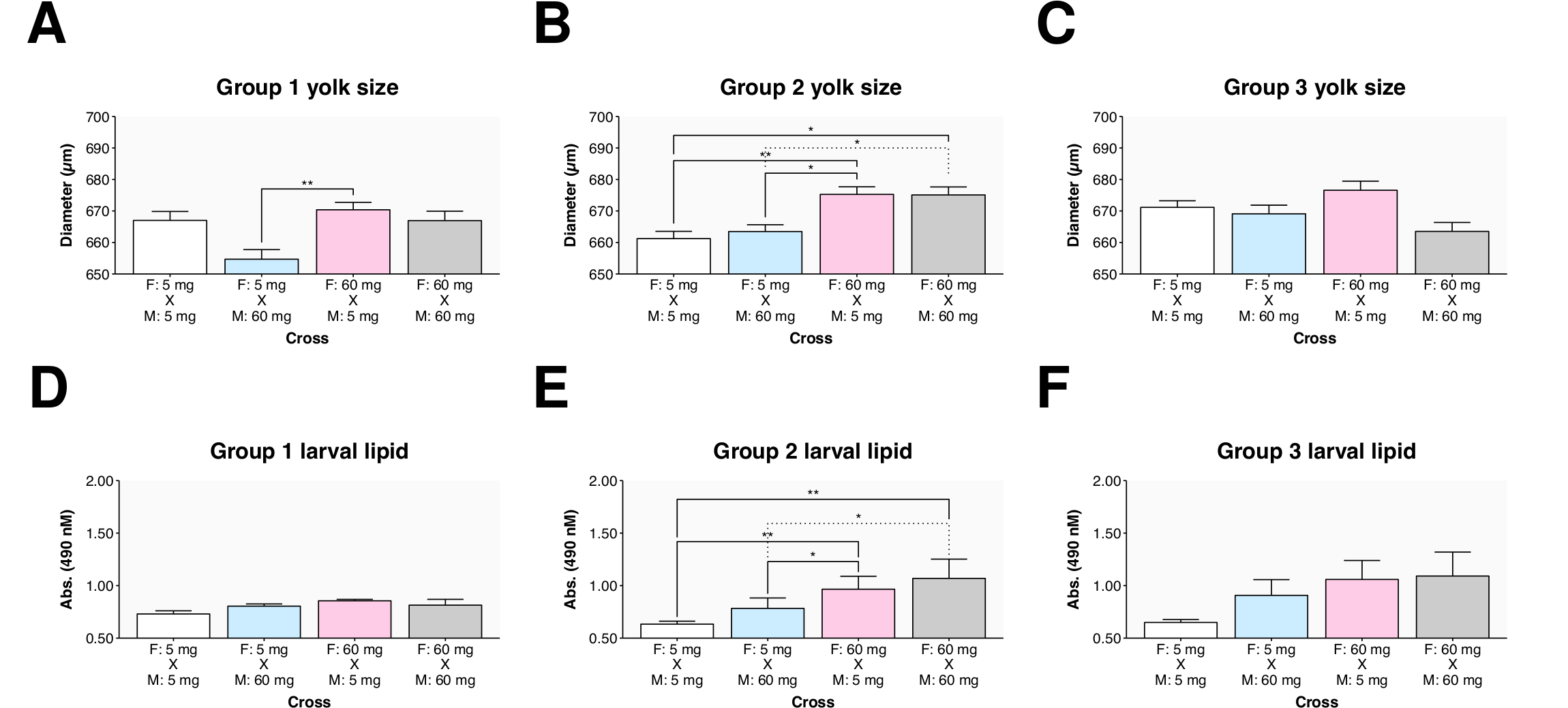

Supplement: S7 Fig — (A-C) The diameter of the egg yolk for the fertilized eggs produced from incrosses within the 5 and 60 mg treatment arm and the outcrosses between them. Values represent the mean ± SEM for 10 individual eggs from each cross from three tanks within each group over four spawning experiments. (D-F) The amount of lipid present at 5 dpf, measured by Oil Red O absorbance at 490 nm. Values represent the mean ± SEM for pools of 30 larvae from each cross from three tanks within each group over three spawning experiments. Statistically significant differences are noted as * p ≤ 0.05 and ** p ≤ 0.01. (TIF) [file pone.0166394.s007.tif]
