# Supplementary material for: Dietary Intake Influences Adult Fertility and Offspring Fitness in Zebrafish
Source: PLoS One. 2016 Nov 21;11(11):e0166394. doi: 10.1371/journal.pone.0166394 (PMC5117665; doi:10.1371/journal.pone.0166394)
Supplement: S1 File — (DOC) [file pone.0166394.s008.doc]

**Supporting Information**

**Supporting methods**

**Adult lipid**

Once the post-diet fertility measurements had been made, the experimental fish were euthanized by hypothermia using incubation in an ice-slurry. Three males and females from each tank were frozen whole in ethanol that had been super-cooled in liquid nitrogen. Serial sections were prepared on a cryostat and stained with Oil Red O (Otago Histology Services Unit, University of Otago). Following high-resolution imaging using the Aperio Digital Slide Scanning System the area of Oil Red O staining was calculated from the best quality section. The remaining fish were dissected in order to measure the weight of the brains and gonad tissue.

**Embryo measurements**

The resulting embryos from each spawning experiment were photographed at 6 hours post fertilization to measure the size of the chorion and the yolk. At 5 dpf, lipid levels were assessed using Oil Red O (Sigma cat. no. O0625). Larvae were fixed in 4% paraformaldehyde for one hour, washed with water, equilibrated with 60% isopropanol, and then stained with 0.6% Oil Red O solution for one hour. The larvae were then washed with water three times before the stain was extracted with 100% isopropanol. The amount of staining was quantified by measuring the absorbance at 500 nm on a plate reader. Pairwise *t*-tests were used to compare the yolk size and lipid staining between embryos.

**Supporting figures**

**S1 Fig. Phenotypic changes resulting from nutrient availability.** Gross phenotypic changes in all three of the groups used in the study. (A-C) Food consumption over the eight weeks of the dietary intervention for the 5 mg arm (meal 1) and the 60 mg arm (meals 1, 2, and 3). The number of *Artemia* remaining following feeding is given as a percentage of the number dispensed to each tank, note the scale begins at 85%. (D-F) The BMI derived from the weights and lengths of every fish in the 5 and 60 mg treatment arms before and after the dietary intervention. The statistical differences are noted between the treatment arms (solid lines) and within each treatment arm (dotted lines), before and after the diet. (G-I) The total distance travelled in 30 seconds of swimming for the populations of fish in the 5 and 60 mg treatment arms at the start (week 1) and the end (week 8) of the diet. Values represent the mean ± SEM from three tanks within each group. Statistically significant differences are noted as * p ≤ 0.05, ** p ≤ 0.01, *** p ≤ 0.001, and **** p ≤ 0.0001.

**S2 Fig. Reproductive consequences of dietary intake.** Fertility changes in all three of the groups used in the study. The breeding success (A-C), clutch size (D-F), and fertilization rate (G-I) is given for each group. Using incrosses within the 5 mg treatment arm (F: 5 mg x M: 5 mg) as the reference for comparison, the effect of the 60 mg treatment was observed using incrosses (F: 60 mg x M: 60 mg) and outcrosses of males (F: 5 mg x M: 60 mg) and females (F: 60 mg x M: 5 mg). The values given represent the odds ratio (A-C), rate ratio (D-F), or incidence rate ratio (G-I) from an average of three pairs from each of three tanks in four spawning experiments. Standard errors were calculated with respect to the tank clusters and are presented as a ± 95% CI. Statistically significant differences are noted as * p ≤ 0.05, ** p ≤ 0.01, *** p ≤ 0.001, and **** p ≤ 0.0001.

**S3 Fig. Raw fertility changes with dietary intake.** Fertility changes in all three of the groups used in the study. (A-C) The breeding success is percent of pairs that bred out of those set up. (D-F) The clutch size is the number of eggs produced by a pair that was successful in breeding. (G-I) The fertilization rate is the percent of eggs that had started to develop by 6 hpf out of the total number of eggs counted. Using incrosses within the 5 mg treatment arm (F: 5 mg x M: 5 mg) as the reference for comparison, the effect of the 60 mg treatment was observed using incrosses (F: 60 mg x M: 60 mg) and outcrosses of males (F: 5 mg x M: 60 mg) and females (F: 60 mg x M: 5 mg). Values represent the mean ± SEM for the three tanks in four spawning experiments, for statistical analysis see S2 Fig.

**S4 Fig. Adult lipid.** (B) Representative images of Oil Red O stained sections taken from the indicated region (A, line) during the F0 necropsy. The total ORO area (S1 Table) was determined by setting a color threshold (B, bottom row) based on image hue, saturation, and brightness. The scale bar given is 1 mm and is relevant for all the sections shown.

**S5 Fig. Swimming activity.** The swimming paths of the fish following the dietary is shown for the 5 and 60 mg treatment arms from each group. The distance travelled by each fish was recorded over 30 seconds and each panel shows the tank of fish that had the average swimming distance for the condition it represents.

**S6 Fig. RNA sequencing reads.** The distribution of mapped read numbers is shown (A) as a density plot for the 5 and 60 mg conditions and (B) as a box plot for the replicate samples. FPKM: fragments per kilobase of transcript per million fragments mapped. (C) The differentially expressed genes are shown in terms of the the fold change and significance in each direction. The significance cut-off was –log(*p* = 0.005) = 2.3.

**S7 Fig. Parental diet influences embryo size and lipid composition.** (A-C) The diameter of the egg yolk for the fertilized eggs produced from incrosses within the 5 and 60 mg treatment arm and the outcrosses between them. Values represent the mean ± SEM for 10 individual eggs from each cross from three tanks within each group over four spawning experiments. (D-F) The amount of lipid present at 5 dpf, measured by Oil Red O absorbance at 490 nm. Values represent the mean ± SEM for pools of 30 larvae from each cross from three tanks within each group over three spawning experiments. Statistically significant differences are noted as * p ≤ 0.05 and ** p ≤ 0.01.

**Supporting tables**

**S1 Table. Phenotypic changes resulting from nutrient availability.** Body parameters describing the F0 fish in groups 1, 2, and 3. Standard length, caudal fin length, total RGB, and weight were measured for every fish and the values are given as the mean ± SEM for the three tanks. Gonad and brain weight were measured for two males and two females from each tank and the values are given as the mean ± SEM for the three tanks. Less fish from cohort 1 were available for dissection so this data is absent. Lipid measurements were made from three males and three females from each tank and the values are given as the mean ± SEM for the three tanks.

**S2 Table. Differentially expressed genes in the eggs.** The 1,630 genes found by RNA-seq to have significantly different expression in the eggs of the 5 and 60 mg females of group 3. Each gene is identified by the gene_id, gene_symbol, and chr_locus. The expression of each gene in the 5 mg and 60 mg samples is given as the FPKM in value_1 and value_2, respectively. The direction and degree of the differential expression is given as the log2(fold_change). The statistical significance of the difference is indicated by the test_stat, p_value, and q_value. Only the significantly altered genes are provided, the significance cut-off was *p* = 0.005.

**S3 Table. Biological processes downregulated in eggs by the 60 mg diet.** Transcripts significantly downregulated by the 60 mg diet (upregulated by the 5 mg diet) were analysed for gene ontology using BiNGO, a Cytoscape plugin [1,2]. Provided in the table is the GO-ID, a description of the biological process, the statistical significance, the number of genes included in the category, and the gene names.

**S4 Table. Biological processes upregulated in eggs by the 60 mg diet.** Transcripts significantly upregulated by the 60 mg diet (downregulated by the 5 mg diet) were analysed for gene ontology using BiNGO, a Cytoscape plugin [1,2]. Provided in the table is the GO-ID, a description of the biological process, the statistical significance, the number of genes included in the category, and the gene names.

**S5 Table. Genes showing differential expression in zebrafish and mice ovaries.** The list of 21 human orthologs that were significantly upregulated by the 5 mg diet (downregulated by the 60 mg diet) in this study and also found to be upregulated by caloric restriction in the mouse ovary by Sharov et al. [3].

**Supporting references**

1. Maere S, Heymans K, Kuiper M. BiNGO: a Cytoscape plugin to assess overrepresentation of gene ontology categories in biological networks. Bioinformatics. 2005;21: 3448–3449.

2. Cline MS, Smoot M, Cerami E, Kuchinsky A, Landys N, Workman C, et al. Integration of biological networks and gene expression data using Cytoscape. Nat Protoc. 2007;2: 2366–2382.

3. Sharov AA, Falco G, Piao Y, Poosala S, Becker KG, Zonderman AB, et al. Effects of aging and calorie restriction on the global gene expression profiles of mouse testis and ovary. BMC Biol. BioMed Central; 2008;6: 1.
